# Distinct conformational states of SARS-CoV-2 spike protein

**DOI:** 10.1126/science.abd4251

**Published:** 2020-07-21

**Authors:** Yongfei Cai, Jun Zhang, Tianshu Xiao, Hanqin Peng, Sarah M. Sterling, Richard M. Walsh, Shaun Rawson, Sophia Rits-Volloch, Bing Chen

**Affiliations:** 1 Division of Molecular Medicine, Boston Children’s Hospital, Boston, MA 02115, USA.; 2 Department of Pediatrics, Harvard Medical School, Boston, MA 02115, USA.; 3 The Harvard Cryo-EM Center for Structural Biology, Harvard Medical School, Boston, MA 02115, USA.; 4 Department of Biological Chemistry and Molecular Pharmacology, Blavatnik Institute, Harvard Medical School, Boston, MA 02115, USA.; 5 SBGrid Consortium, Harvard Medical School, Boston, MA 02115, USA.

## Abstract

Intervention strategies are urgently needed to control the SARS-CoV-2 (severe acute respiratory syndrome coronavirus 2) pandemic. The trimeric viral spike (S) protein catalyzes fusion between viral and target cell membranes to initiate infection. Here we report two cryo-EM structures, derived from a preparation of the full-length S protein, representing its prefusion (2.9Å resolution) and postfusion (3.0Å resolution) conformations, respectively. The spontaneous transition to the postfusion state is independent of target cells. The prefusion trimer has three receptor-binding domains clamped down by a segment adjacent to the fusion peptide. The postfusion structure is strategically decorated by N-linked glycans, suggesting possible protective roles against host immune responses and harsh external conditions. These findings advance our understanding of SARS-CoV-2 entry and may guide development of vaccines and therapeutics.

The current coronavirus pandemic is having devastating social and economic consequences. Coronaviruses (CoVs) are enveloped positive-stranded RNA viruses. They include severe acute respiratory syndrome (SARS) and Middle East respiratory syndrome (MERS), both with significant fatalities (*1*–*3*), as well as several endemic common-cold viruses (*4*). With a large number of similar viruses circulating in bats and camels (*5*–*8*), the possibility of additional outbreaks poses major threats to global public health. The current disease, COVID-19 (coronavirus disease 2019), caused by a new virus SARS-CoV-2 (*9*), has created urgent needs for diagnostics, therapeutics and vaccines. Meeting these needs requires a deep understanding of the structure-function relationships of viral proteins and relevant host factors.

For all enveloped viruses, membrane fusion is a key early step for entering host cells and establishing infection (*10*). Although an energetically favorable process, membrane fusion has high kinetic barriers when two membranes approach each other, mainly due to repulsive hydration forces (*11*, *12*). For viral membrane fusion, free energy to overcome these kinetic barriers comes from refolding of virus-encoded fusion proteins from a primed, metastable prefusion conformational state to a stable, postfusion state (*13*–*15*). The fusion protein for CoV is its spike (S) protein that decorates the virion surface as an extensive crown (hence, “corona”). The protein also induces neutralizing antibody responses and is therefore an important target for vaccine development (*16*). The S protein is a heavily glycosylated type I membrane protein anchored in the viral membrane. It is first produced as a precursor that trimerizes and is thought to be cleaved by a furin-like protease into two fragments: the receptor-binding fragment S1 and the fusion fragment S2 (Fig. 1A) (*17*). Binding through the receptor-binding domain (RBD) in S1 to a host cell receptor (e.i., angiotensin converting enzyme 2 (ACE2) for both SARS-CoV and SARS-CoV-2) and further proteolytic cleavage at a second site in S2 (S2’ site), by a serine protease TMPRSS2 (*18*) or endosomal cysteine proteases cathepsins B and L (CatB/L), are believed to trigger dissociation of S1 and irreversible refolding of S2 into a postfusion conformation – a trimeric hairpin structure formed by heptad repeat 1 (HR1) and heptad repeat 2 (HR2) (*19*, *20*). These large structural rearrangements bring together the viral and cellular membranes, ultimately leading to fusion of the two bilayers.

**Fig. 1:**
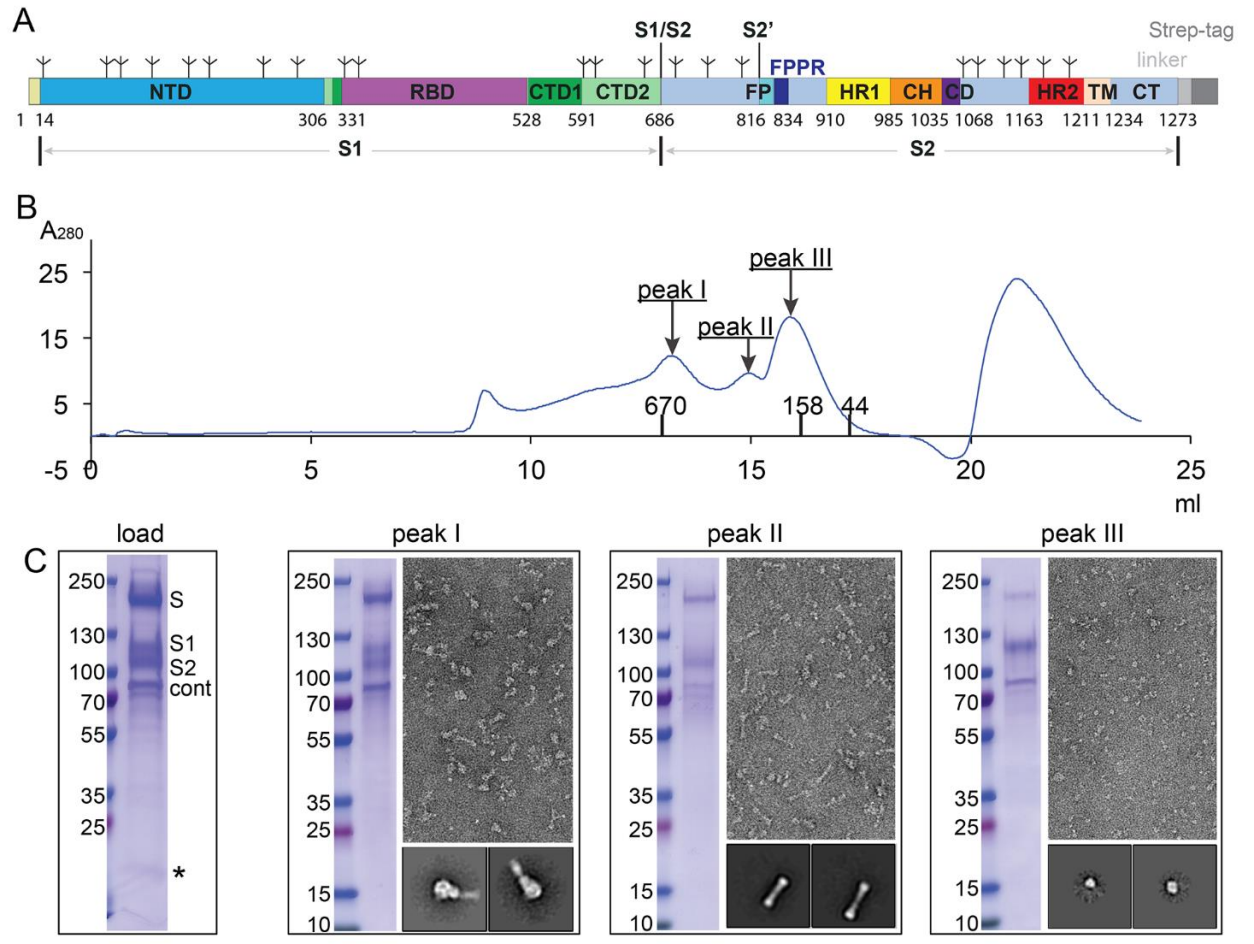
Preparation of a full-length SARS-CoV-2 spike protein. (**A**) Schematic representation of the expression construct of full-length SARS-CoV-2 spike (S) protein. Segments of S1 and S2 include: NTD, N-terminal domain; RBD, receptor-binding domain; CTD1, C-terminal domain 1; CTD2, C-terminal domain 2; S1/S2, S1/S2 cleavage site; S2’, S2’ cleavage site; FP, fusion peptide; FPPR, fusion peptide proximal region; HR1, heptad repeat 1; CH, central helix region; CD, connector domain; HR2, heptad repeat 2; TM, transmembrane anchor; CT, cytoplasmic tail; and tree-like symbols for glycans. A strep-tag was fused to the C terminus of S protein by a flexible linker. (**B**) The purified S protein was resolved by gel-filtration chromatography on a Superose 6 column in the presence of detergent NP-40. The molecular weight standards include thyoglobulin (670 kDa), γ-globulin (158 kDa) and ovalbumin (44 kDa). Three major peaks (peak I-III) contain the S protein. (**C**) Load sample and peak fractions from (B) were analyzed by Coomassie stained SDS-PAGE. Labeled bands were confirmed by Western blot (S, S1 and S2) or protein sequencing (S2 and Cont; S and S1 bands did not gave any meaningful results probably due to a blocked N terminus). Cont, copurified contaminating protein, identified as endoplasmic reticulum chaperone BiP precursor by N-terminal sequencing. *, a putative S1/S2-S2’ fragment. Representative images and 2D averages by negative stain EM of three peak fractions are also shown. The box size of 2D averages is ~510Å.

Since the first genome sequence of SARS-CoV-2 was released (*21*), several structures have been reported for S protein complexes, including the ectodomain stabilized in the prefusion conformation (*22*–*24*) and RBD-ACE2 complexes (*25*–*28*) (fig. S1), building upon the previous success of the structural biology of S proteins from other CoVs (*20*). In the stabilized S ectodomain, S1 folds into four domains - NTD (N-terminal domain), RBD, and two CTDs (C-terminal domains) and protects the prefusion conformation of S2 in which HR1 bends back toward the viral membrane (fig. S1, A and B). The RBD samples two distinct conformations – “up” representing a receptor-accessible state and “down” representing a receptor-inaccessible state. Structures representing the postfusion state of S2 from mouse hepatitis virus (fig. S1E) and a lower-resolution one from SARS-CoV (fig. S1F), suggest how the structural rearrangements of S2 proceed to promote membrane fusion and viral entry (*29*, *30*). Comparison of the pre- and post-fusion states reveals that HR1 undergoes a “jack-knife” transition that can insert the fusion peptide (FP) into the target cell membrane. Folding back of HR2 places the FP and transmembrane (TM) segments at the same end of the molecule, causing the membranes with which they interact to bend toward each other, effectively leading to membrane fusion. In the previous structures, the regions near the viral membrane are either not present or disordered, and yet they all appear to play critical structural and functional roles (*31*–*35*).

To gain further insight, we aimed to determine the pre and post fusion states of full-length wild-type S protein of SARS-CoV-2.

## Results

### Purification of intact S protein

To produce a functional SARS-CoV-2 S protein, we transfected HEK293 cells with an expression construct of a full-length wildtype S sequence with a C-terminal strep-tag (Fig. 1A). These cells fused efficiently with cells transfected with an intact human ACE2 construct, even without addition of any extra proteases (fig. S2), suggesting that the S protein expressed on the cell surfaces is fully functional for membrane fusion. The fusion efficiency was not affected by the C-terminal strep-tag. To purify the full-length S protein, we lysed the cells and solubilized all membrane-bound proteins in 1% detergent NP-40. The strep-tagged S protein was then captured on strep-tactin resin in 0.3% NP-40. The purified S protein eluted from a size-exclusion column as three distinct peaks in 0.02% NP-40 (Fig. 1B). Analysis by Coomassie-stained SDS-PAGE (Fig. 1C) showed that peak 1 contained both the uncleaved S precursor and the cleaved S1/S2 complex; peak 2 had primarily the cleaved but dissociated S2 fragment; and peak 3 included mainly the dissociated S1 fragment, as judged by N-terminal sequencing and Western blot (fig. S3). This was confirmed by negative stain EM (Fig. 1C). Peak 1 showed the strongest binding to soluble ACE2, comparable to that for the purified soluble S ectodomain trimer, while peak 2 showed the weakest binding, since it contained mainly the S2 fragment (fig. S4). While the cleavage at the S1/S2 (furin) site is clearly demonstrated by protein sequencing of the N terminus of the S2 fragment in peak 2, cleavage at the S2’ site is not obvious. We observed in some preparations a band around 20 kDa, a size expected for the S1/S2-S2’ fragment (Fig. 1C). We obtained a similar gel filtration profile when another detergent (DDM) was used to solubilize the S protein (fig. S5), suggesting that the S protein dissociation during gel filtration chromatography is not triggered by any specific detergent. We also identified a major contaminating protein in the preparation as endoplasmic reticulum chaperone BiP precursor (*36*), which may have a role in facilitating S protein folding.

### Cryo-EM structure determination

Cryo-EM images were acquired with selected grids prepared from all three peaks, on a Titan Krios electron microscope operated at 300 keV and equipped with a BioQuantum energy filter and a Gatan K3 direct electron detector. We used RELION (*37*) for particle picking, two-dimensional (2D) classification, three dimensional (3D) classification and refinement. Structure determination was performed by rounds of 3D classification, refinement and masked local refinement, as described in the supplementary materials. The final resolution was 2.9Å for the prefusion S protein; 3.0Å for the S2 in the postfusion conformation (figs. S6 to S9).

### Structure of the prefusion S trimer

**Fig. 2:**
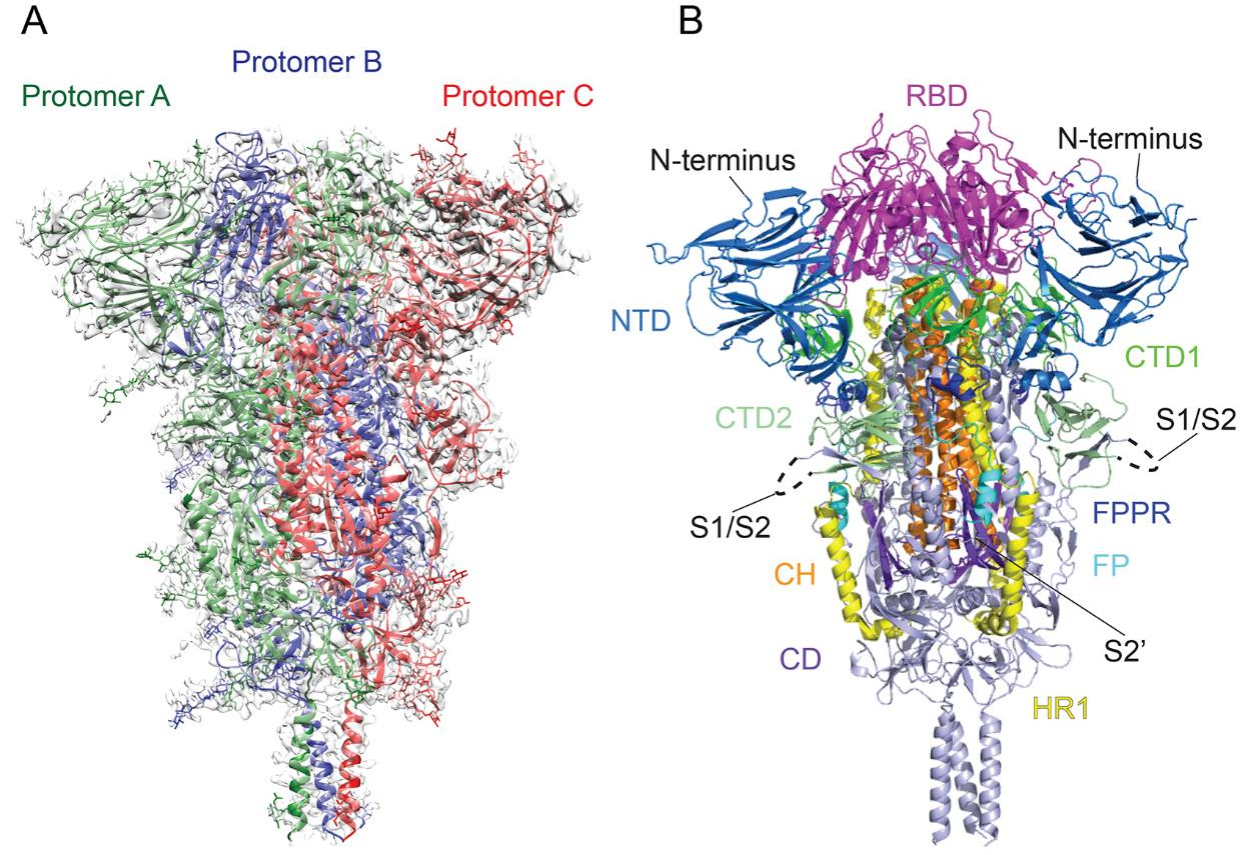
Cryo-EM structure of the SARS-CoV-2 S protein in the prefusion conformation. (**A**) The structure of the S trimer was modeled based on a 2.9Å density map. Three protomers (A, B, and C) are colored in green, blue and red, respectively. (**B**) Overall structure of S protein in the prefusion conformation shown in ribbon representation. Various structural components in the color scheme shown in Fig. 1A include NTD, N-terminal domain; RBD, receptor-binding domain; CTD1, C-terminal domain 1; CTD2, C-terminal domain 2; FP, fusion peptide; FPPR, fusion peptide proximal region; HR1, heptad repeat 1; CH, central helix region; and CD, connector domain. N terminus, S1/S2 cleavage site and S2’ cleavage site are indicated.

The overall architecture of the full-length S protein in the prefusion conformation is very similar to the published structures of a soluble S trimer stabilized by a C-terminal foldon trimerization tag and two proline substitutions at the boundary between HR1 and the central helix (CH) in S2 (fig. S1) (*22*, *23*). In our new structure, the N terminus, several peripheral loops and glycans that were invisible in the soluble trimer structures are ordered (Fig. 2, A and B, and fig. S10A). As described previously, the four domains of the S1 fragment, NTD, RBD, CTD1 and CTD2, wrap around the three-fold axis, covering the S2 fragment underneath. The furin cleavage site at the S1/S2 boundary is in a surface-exposed and disordered loop (Fig. 2B), so it is unclear whether this structure represents the uncleaved or cleaved trimer, although the sample clearly contains both forms (Fig. 1C). Likewise, the S2 fragment has a conformation nearly identical to that in the previous trimer structures, with most of the polypeptide chain packed around a central three-stranded coiled coil formed by CH, including the connector domain (CD), which links CH and the C-terminal HR2 through an additional linker region. A difference between our structure and the published trimer structures is that a ~25-residue segment in S2 immediately downstream of the fusion peptide is ordered. The segments, HR2, TM and CT, not observed in previous structures, are still not visible.

**Fig. 3:**
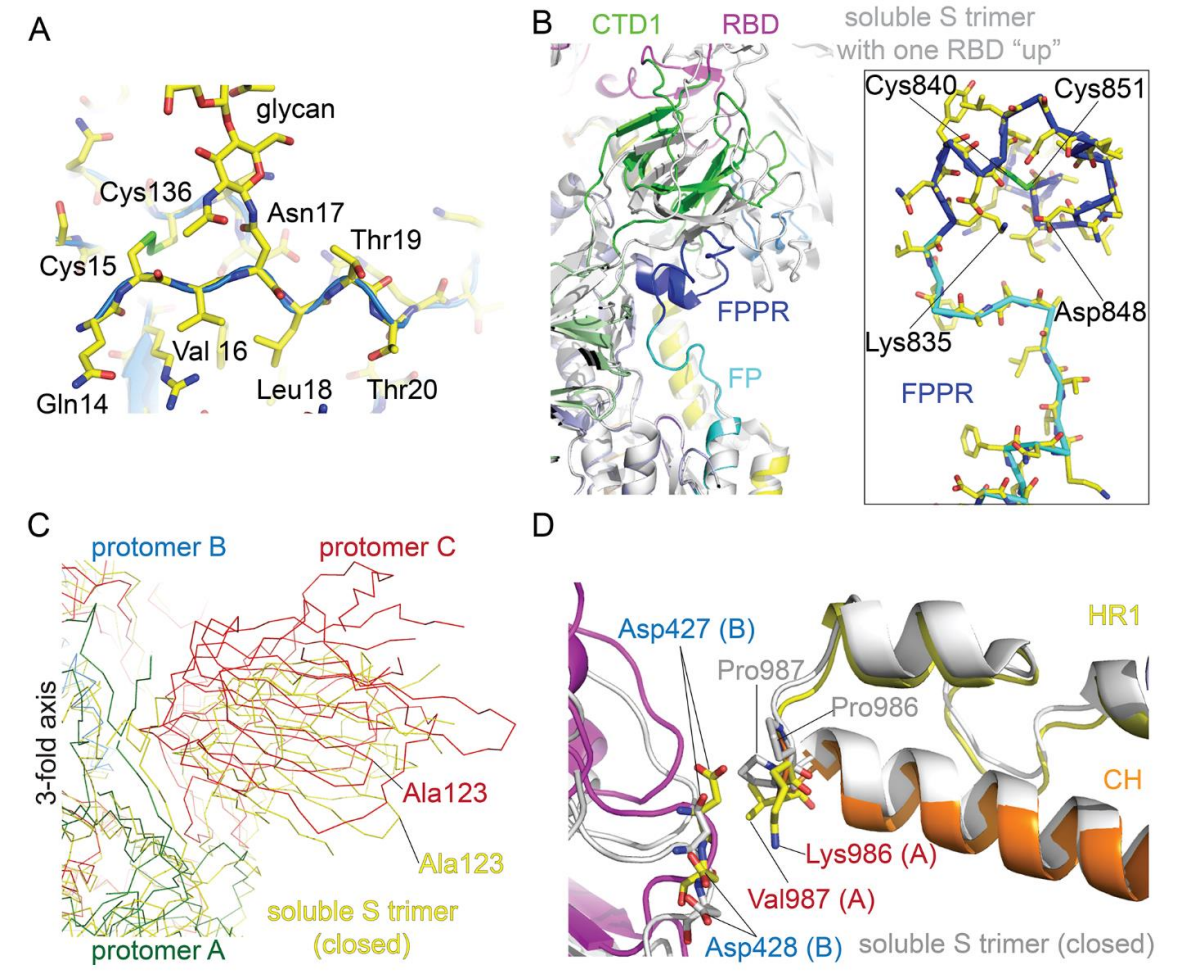
Selected new features of the SARS-CoV-2 prefusion S trimer. (**A**) N-terminal segment of S protein. The N terminus is at residue Gln14 after cleavage of the signal peptide. Cys15 forms a disulfide bond with Cys136. We observed good density for the N-linked glycan at Asn17. (**B**) A segment immediately downstream of the fusion peptide, while disordered in the stabilized soluble S ectodomain trimer structure, forms a tightly packed structure, designated FPPR for the fusion peptide proximal region, abutting CTD1. The newly identified FPPR structure would clash with CTD1 in the RBD up conformation. Various domains are shown in the color scheme in Fig. 2B. The structure of the soluble S trimer with one RBD in the up conformation (PDB ID: 6vyb) is shown in gray. In the box, a close-up view of the FPPR with adjacent fusion peptide in both surface representation and stick model. (**C**) The SARS-CoV-2 prefusion S trimer, viewed along the threefold axis, is superposed on the structure of the stabilized soluble S ectodomain trimer in the closed conformation with all three RBDs in the down conformation (PDB ID: 6vxx). While the S2 region is well aligned, there is a significant shift (e.g., ~12Å between two Ala123 residues) in S1. (**D**) Impact of the proline mutations introduced at residues 986 and 987 to stabilize the prefusion conformation. K986P mutation removes a salt bridge between Lys986 of one protomer and either Asp427 or Asp428 of another protomer in the trimer interface.

Several features are different between our structure and the previously described prefusion conformations. First, the N terminus in our structure is ordered and adopts a conformation similar to that in SARS-CoV, including a disulfide bond (Cys15-Cys136) and a N-linked glycan at Asn17 (Fig. 3A) (*38*). It would be important to confirm whether this region is unfolded with no disulfide bond in the stabilized soluble constructs or folded and simply poorly defined by density, despite a disulfide bond, particularly if they are widely used for vaccine studies.

Second, another disulfide containing segment (residues 828-853), immediately downstream of the fusion peptide is also absent from the structures of the soluble ectodomain, but ordered in our structure (Fig. 3B). We designate it as the fusion-peptide proximal region (FPPR). The FPPR is disordered in both the closed and RBD-up conformations of the stabilized soluble S trimer. In our full-length structure, it packs rather tightly around an internal disulfide bond between Cys840 and Cys851, further reinforced by a salt bridge between Lys835 and Asp848, as well as by an extensive hydrogen bond network. When compared with the RBD-up conformation by superposition of the rest of S2, the FPPR clashes with CTD1, which rotates outwards with the RBD in the flipping-up transition. Thus, a structured FPPR, abutting the opposite side of CTD1 from the RBD, appears to help clamp down the RBD and stabilize the closed conformation of the S trimer. It is not obvious why the FPPR is also not visible in the published, closed S ectodomain structure with all three RBDs in the down conformation (*23*). Our structure of the full-length S protein suggests that CTD1 is a structural relay between RBD and FPPR that can sense the displacement on either side. The latter is directly connected to the fusion peptide. Lack of a structured FPPR in the stabilized, soluble S trimer may explain why the RBD-up conformation is readily detected in that preparation. In addition, a D614G mutation, identified in recent SARS-CoV-2 isolates, has been suggested to lead to more efficient entry (*39*, *40*). D614 forms a salt bridge with K854 in the FPPR (fig. S10B), supporting a functional role of the FPPR in membrane fusion. In the 3D classification of our prefusion particles from two independent data sets, only one subclass with an RBD flipped up was observed (fig. S6), suggesting that the RBD-up conformation is relatively rare in our full-length S preparation. The map for this subclass was refined to 4.7Å without C3 symmetry and we could not model the FPPR. The FPPR is ordered in all other maps that are refined to 3.5Å or higher resolution.

When we aligned our full-length structure with the soluble S trimer structure by the S2 portion, the three S1 subunits in the soluble trimer structure move outwards away from the three-fold axis, up to ~12Å in peripheral areas (Fig. 3C and fig. S11), suggesting the full length S trimer is more tightly packed among the three protomers than the mutated soluble trimer. Examining the region near the proline mutations between HR1 and CH, we found that the K986P mutation appeared to eliminate a salt bridge between Lys986 in one protomer and either Asp 427 or Asp428 in another protomer; thus, the mutation could create a net charge (three for one trimer) inside the trimer interface. This may explain why the soluble trimer with the PP mutation has a looser structure than the full-length S with wildtype sequence. Whether this loosening leads to disordered FPPRs in the closed trimer will require additional experimental evidence. However, the proline mutations, designed to destabilize the postfusion conformation and strengthen the prefusion structure, may also impact the prefusion structure.

### Structure of the postfusion S2 trimer

**Fig. 4:**
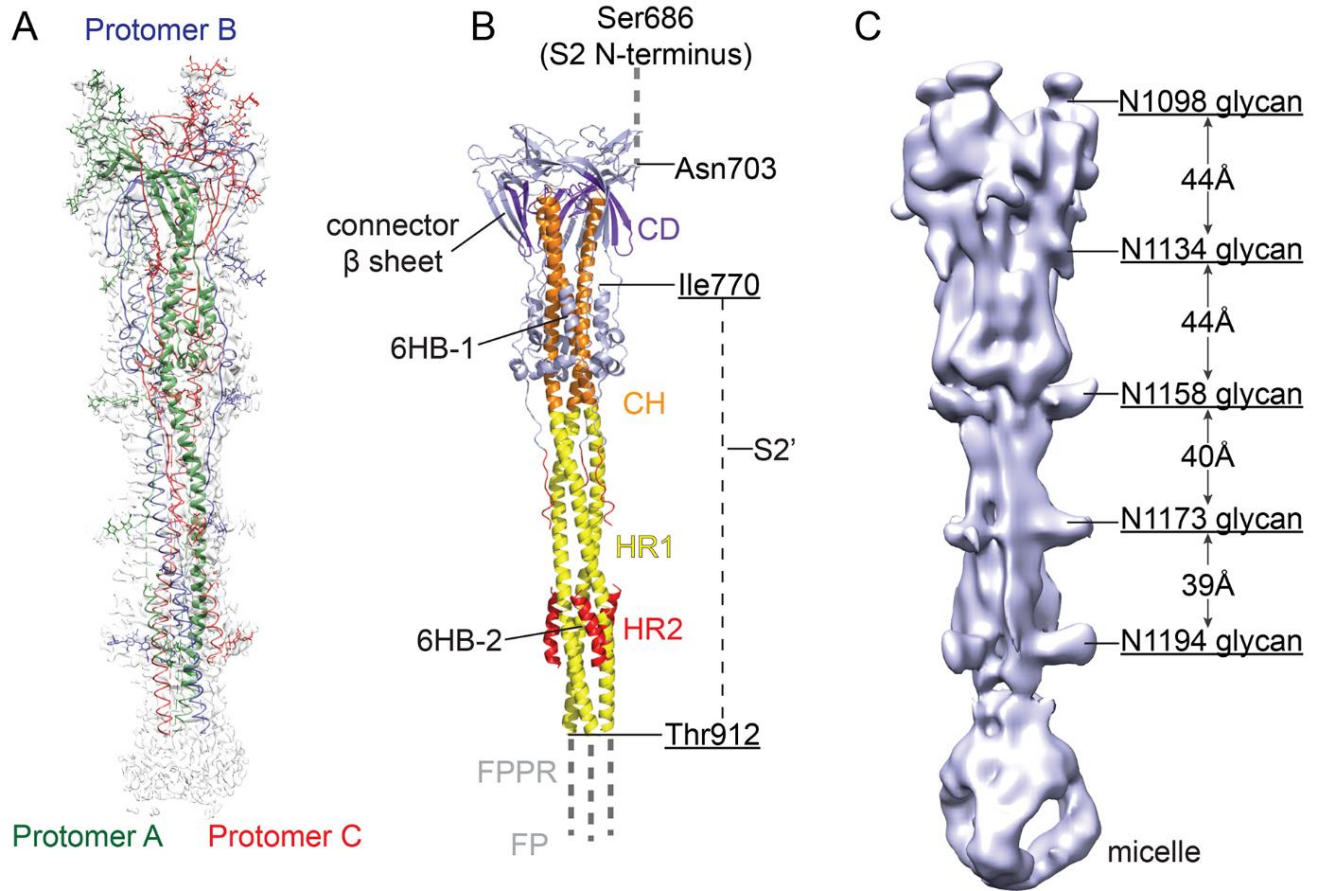
Cryo-EM structure of the SARS-CoV-2 S2 in the postfusion conformation. (**A**) The structure of the S2 trimer was modeled based on a 3.3Å density map. Three protomers (A, B, and C) are colored in green, blue and red, respectively. (**B**) Overall structure of the S2 trimer in the postfusion conformation shown in ribbon diagram. Various structural components in the color scheme shown in Fig. 1A include HR1, heptad repeat 1; CH, central helix region; CD, connector domain; and HR1, heptad repeat 2. The S2’ cleavage site is in a disordered loop between Ile770 and Thr912. Possible locations of the S2 N terminus (S1/S2 cleavage site), the FP and FPPR are also indicated. (**C**) A low-resolution map showing the density pattern for 5 N-linked glycans, with almost equal spacing along the long axis.

3D reconstruction of the sample from peak 2 yielded a postfusion structure of the S2 trimer, shown in Fig. 4A. The overall architecture of the SARS-CoV-2 S2 in the postfusion conformation is nearly identical to that of the published structure derived from the S2 ectodomain of mouse hepatitis virus (MHV) produced in insect cells (fig. S1) (*29*). In the structure, HR1 and CH form an unusually long central three-stranded coiled coil (~180Å). The connector domain, together with a segment (residues 718-729) in the S1/S2-S2’ fragment, form a three-stranded β sheet, which is invariant between the prefusion and postfusion structures. In the postfusion state, residues 1127-1135 join the connector β sheet to expand it into four strands, while projecting the C-terminal HR2 toward the viral membrane. Another segment (residues 737-769) in the S1/S2-S2’ fragment makes up three helical regions locked by two disulfide bonds that pack against the groove of the CH part of the coiled coil to form a short six helix bundle structure (6HB-1 in Fig. 4B). It is unclear whether the S’2 site is cleaved because it is in a disordered region spanning 142 residues (Fig. 4B), as in the MHV S2 structure. Nevertheless, the S1/S2-S2’ fragment is an integral part of the postfusion structure and would not dissociate, regardless of cleavage at the S2’ site. The N-terminal region of HR2 adopts a one-turn helical conformation and also packs against the groove of the HR1 coiled-coil; the C-terminal region of HR2 forms a longer helix that makes up the second six-helix bundle structure with the rest of the HR1 coiled-coil (6HB-2 in Fig. 4B). Thus, the long central coiled-coil is reinforced multiple times along its long axis, making it a very rigid structure, as evident even from 2D class averages of particles in the cryo images (fig. S8).

A striking feature of the postfusion S2 is its surface decoration by N-linked glycans (Fig. 4C), also visible in the 2D class averages (fig. S8). Five glycans at residues Asn1098, Asn1134, Asn1158, Asn1173 and Asn1194 are positioned along the long axis with a regular spacing with four of them aligned on the same side of the trimer. If these glycosylation sites are fully occupied by branched sugars, they may shield most surfaces of the postfusion S2 trimer. A similar pattern has been recently described in a paper posted in ChinaXiv (http://www.chinaxiv.org/user/download.htm?id=30394) for a SARS-CoV S2 preparation derived from a soluble S ectodomain construct produced in insect cells and triggered by proteolysis and low pH. The reason for this decoration is unclear given that a postfusion structure has accomplished its mission, and should not need to be concealed from the immune system.

Peak 3 contains primarily the dissociated monomeric S1 fragment, which has the smallest size (~100 kDa) and shows the lowest contrast in cryo grids of the three particle types we describe. We carried out a preliminary 3D reconstruction analysis (fig. S12), further confirming its identity.

## Discussion

### Architecture of S protein on the surface of SARS-CoV-2 virion

**Fig. 5:**
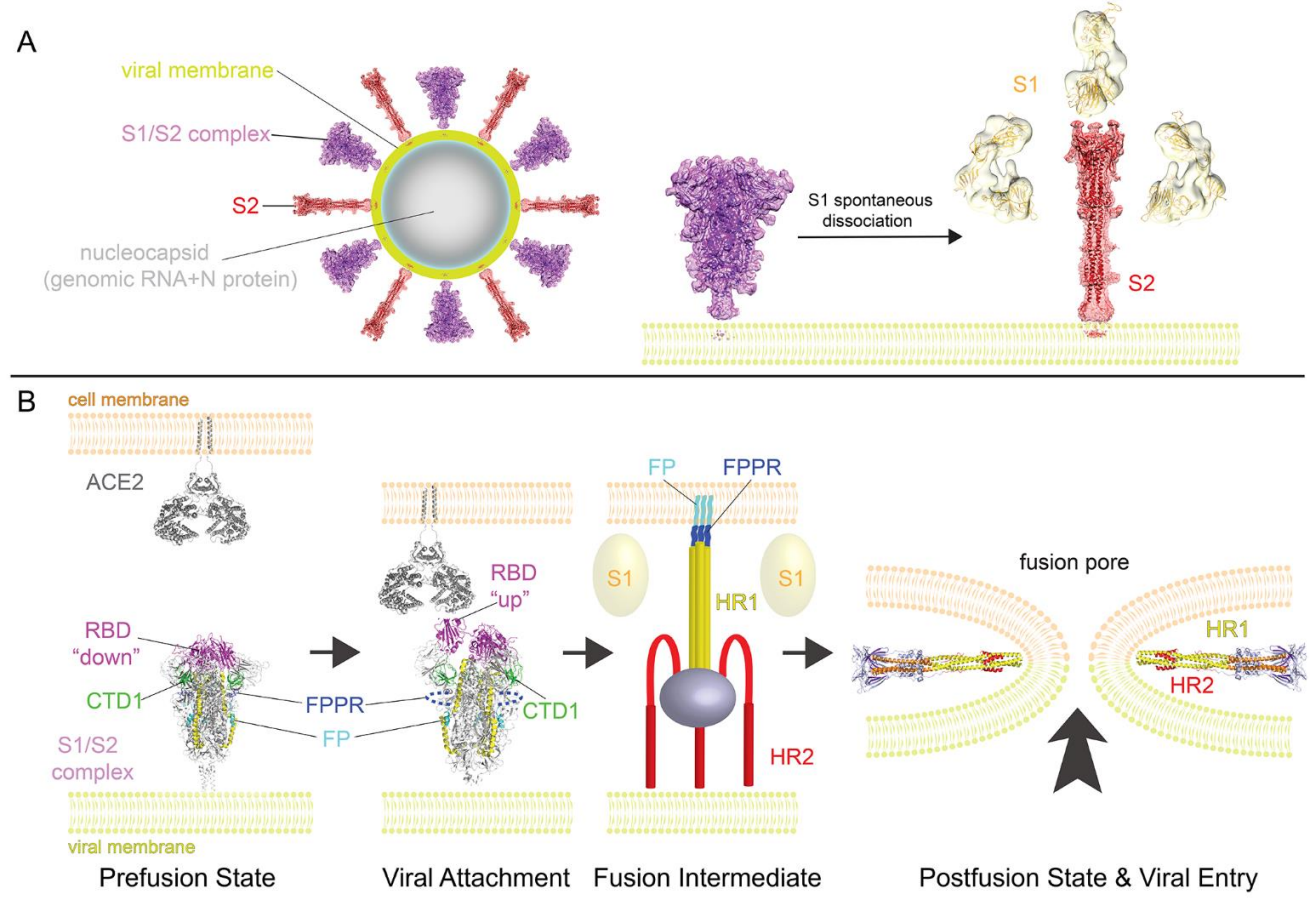
A model for structural rearrangements of SARS-Cov-2 S protein. (**A**) Structural changes independent of a target cell. We suggest that both the prefusion and postfusion spikes are present on the surface of mature virion and the ratio between them may vary (diagram of virion). The postfusion spikes on the virion are formed by S2 after S1 dissociates in the absence of ACE2. (**B**) ACE2-dependent structural rearrangements. Structural transition from the prefusion to postfusion conformation inducing membrane fusion likely proceeds stepwise as follows: 1) FPPR clamps down RBD through CTD1 in the prefusion S trimer (this study), but it occasionally flips out of position and allows an RBD to sample the up conformation (PDB ID: 6vyb). 2) RBD binding to ACE2 (PBD ID: 6m17) creates a flexible FPPR that enables exposure of the S2’ cleavage site immediately upstream of the adjacent fusion peptide (FP). Cleavage at the S2’ site, and perhaps also the S1/S2 site, releases the structural constraints on the fusion peptide and initiates a cascade of refolding events in S2, probably accompanied by complete dissociation of S1. 3) Formation of the long central three-stranded coiled-coil and folding back of HR2. 4) Formation of the postfusion structure of S2 (this study) that brings the two membranes together, facilitating formation of a fusion pore and viral entry.

The fact that the cleaved S1/S2 complex dissociates in the absence of ACE2 and that the S2 fragment adopts a postfusion conformation under mild detergent conditions, suggesting that the kinetic barrier for the conformational transition relevant to viral entry is surprisingly low for this S protein. Whether or not this observation relates directly to efficient membrane fusion or infection is unclear. Nevertheless, it is noteworthy that the postfusion S2 trimer not only has a very stable and rigid structure, but also that it is strategically decorated with N-linked glycans along its long axis, as if under selective pressure for functions other than the membrane fusion process. Although some have suggested that viral fusion proteins may further oligomerize in their postfusion conformation to facilitate fusion pore formation (*41*), the protruding surface glycans of the SARS-CoV-2 S2 make this scenario unlikely. A more plausible possibility is a protective role that the S2 postfusion structure could play if it is also present on the surface of an infectious and mature virion. It may induce nonneutralizing antibody responses to evade the host immune system; it may also shield the more vulnerable prefusion S1/S2 trimers under conditions outside the host by decorating the viral surface with interspersed rigid spikes (Fig. 5A). Several recent reports have provided some evidence supporting this possibility. First, EM images of a β-propiolactone inactivated SARS-CoV-2 virus preparation, purified by a potassium tartrate-glycerol density gradient, appeared to have lost all S1 subunits, leaving only the postfusion S2 on the virion surfaces (*42*). Likewise, EM images of a β-propiolactone inactivated SARS-CoV-2 virus vaccine candidate (PiCoVacc) also showed needle-like spikes on its surfaces (*43*). Second, spontaneous shedding of SARS-CoV-2 S1 from pseudoviruses in absence of ACE2 has been reported (*39*). Third, binding antibodies against S2 are readily detectable in COVID-19 patients (*44*), suggesting S2 is more exposed to the host immune system than indicated by the unprotected surfaces on the prefusion structures (*22*, *23*) (Fig. 2). We therefore suggest that postfusion S2 trimers may have a protective function by constituting part of the crown on the surface of mature and infectious SARS-CoV-2 virion (Fig. 5). The postfusion S2 spikes are probably formed after spontaneous dissociation of S1, independent of the target cells.

### Membrane fusion

We identify a structure near the fusion peptide – the fusion peptide proximal region (FPPR), which may play a critical role in the fusogenic structural rearrangements of S protein. There appears to be crosstalk between the RBD and the FPPR, mediated by CTD1, as a structured FPPR clamps down the RBD while an RBD-up conformation disorders the FPPR. Moreover, the FPPR is close to the S1/S2 boundary and the S2’ cleavage site, and thus might be the center of activities relevant to conformational changes in S. One possibility is that one FPPR occasionally flips out of position due to intrinsic protein dynamics, allowing the RBDs to sample the up conformation. A fluctuation of this kind would loosen the entire S trimer, as observed in modified soluble S trimer constructs (*22*, *23*). Once an RBD is fixed in the up position by binding to ACE2 on the surface of a target cell, a flexible FPPR may enable exposure of the S2’ cleavage site immediately upstream of the adjacent fusion peptide. The phenotype of the D614G mutation appears to be consistent with the notion that the FPPR is involved in membrane fusion (*39*, *40*). Cleavage at the S2’ site releases the structural constraints on the fusion peptide, which may initiate a cascade of refolding events in S2, including formation of the long central three stranded coiled-coil, folding back of HR2 and ultimately membrane fusion. Cleavage at the S1/S2 site allows complete dissociation of S1, which may also facilitate S2 refolding.

Puzzles regarding membrane fusion remain, as the regions near the viral membrane are still not visible in the reconstructions. Yet these regions all play critical structural and functional roles. For example, the conserved hydrophobic region immediately preceding the TM domain, and possibly the TM itself, have been shown to be crucial for S protein trimerization and membrane fusion (*31*). The cytoplasmic tail, containing a palmitoylated cysteine-rich region, is believed to be involved in viral assembly and cell-cell fusion (*32*–*35*). Whether other viral proteins, such M protein, may help stabilize the spike by interacting with the HR2 remains an interesting question. Thus, we still need a high-resolution structure of an intact S protein in the context of the membrane and other viral components to answer the various open questions.

### Considerations for vaccine development

A safe and effective vaccine is the primary medical option to reduce or eliminate the threat posed by SARS-CoV-2. The first round of vaccine candidates with various forms of the spike (S) protein of the virus are passing rapidly through preclinical studies in animal models and clinical trials in humans. Our study raises several potential concerns about the current vaccine strategies. First, vaccines using the full-length wildtype sequence of S protein may produce the various forms in vivo that we have observed here. The postfusion conformations could expose immunodominant, nonneutralizing epitopes that distract the host immune system, as documented for other viruses, such as HIV-1 and RSV (*45*, *46*). Second, the approach to stabilize the prefusion conformation by introducing proline mutations at residues 986 and 987 may not be optimal, as the K986P mutation may break a salt bridge between protomers that contributes to the trimer stability. The resulting S trimer structure with a relaxed apex may induce antibodies that could not efficiently recognize S trimer spikes on the virus, although it may be more effective in inducing anti-RBD neutralizing responses than the closed form. Third, in light of the possibility that the postfusion S2 is present on infectious virions, vaccines using β-propiolactone inactivated viruses may require additional quality control tests. Although the PiCoVacc appears to provide protection against challenges in nonhuman primates after three immunizations (*43*), it is unclear how to minimize the number of the postfusion S2 trimers to avoid batch variations. Structure-guided immunogen design may be particularly critical if SARS-CoV-2 becomes seasonal and returns with antigenic drift, as do influenza viruses (*47*).

## Supplementary Materials

science.sciencemag.org/cgi/content/full/science.abd4251/DC1 Materials and Methods Figs. S1 to S12 Table S1 References (*48*–*57*) MDAR Reproducibility Checklist
